# A Cylindrical Near-Field Acoustical Holography Method Based on Cylindrical Translation Window Expansion and an Autoencoder Stacked with 3D-CNN Layers

**DOI:** 10.3390/s23084146

**Published:** 2023-04-20

**Authors:** Jiaxuan Wang, Weihan Zhang, Zhifu Zhang, Yizhe Huang

**Affiliations:** 1 State Key Laboratory of Digital Manufacturing and Technology, Huazhong University of Science and Technology, Wuhan 430074, China; wjx@hust.edu.cn (J.W.);; 2 School of Mechanical and Electrical Engineering, Hainan University, Haikou 570228, China; 3 School of Mechanical Engineering, Hubei University of Technology, Wuhan 430068, China

**Keywords:** cylindrical near-field acoustic holography, sparse sampling, translation window, 3D-CNN stacked auto encoder, PGa extrapolation interpolation

## Abstract

The performance of near-field acoustic holography (NAH) with a sparse sampling rate will be affected by spatial aliasing or inverse ill-posed equations. Through a 3D convolution neural network (CNN) and stacked autoencoder framework (CSA), the data-driven CSA-NAH method can solve this problem by utilizing the information from data in each dimension. In this paper, the cylindrical translation window (CTW) is introduced to truncate and roll out the cylindrical image to compensate for the loss of circumferential features at the truncation edge. Combined with the CSA-NAH method, a cylindrical NAH method based on stacked 3D-CNN layers (CS3C) for sparse sampling is proposed, and its feasibility is verified numerically. In addition, the planar NAH method based on the Paulis–Gerchberg extrapolation interpolation algorithm (PGa) is introduced into the cylindrical coordinate system, and compared with the proposed method. The results show that, under the same conditions, the reconstruction error rate of the CS3C-NAH method is reduced by nearly 50%, and the effect is significant.

## 1. Introduction

Acoustic array technology can realize acoustic imaging through certain signal processing methods after measuring acoustic signals through sensors. These technologies include sound intensity, beamforming technology and near-field acoustic holography technology (NAH). The sound intensity method measures the gradient of the sound pressure along the normal direction, so that the radiation power and the direction of the sound intensity can be obtained. The NAH method utilizes the information from surface evanescent waves and the spatial phase to calculate near-field acoustical quantities accurately and efficiently through the discrete spatial Fourier transform. The measurement distance of these two methods is limited to the minimum half-wavelength region. For far-field and high-frequency issues, beamforming technologies may be selected as an alternative. Among them, the NAH method can visualize the sound field with a high level of precision and complete phase information due to the supplementary utilization of the detailed information contained in the evanescent wave components. Furthermore, it is not limited by the Rayleigh criterion, accounting for improvement in the spatial resolution of the reconstructed quantities. The highest spatial resolution of the reconstructed sound field can be one order of magnitude lower than the sound wavelength. As an acoustic inverse imaging method for non-contact measurement, NAH is widely used in the fields of mechanical fault diagnosis [1], noise source diagnosis [2,3], aerospace [4,5] and to address issues concerned with vehicle noise, vibration and harshness (NVH) [6]. NAH theories have evolved; the theory initially proposed is based on spatial Fourier transform (SFT-NAH). The core of it is that the spatial Fourier transform is utilized to transform the complex sound pressure or particle vibration velocity on the measurement surface from the space domain to the wave number domain, which will be processed by appropriate transfer operator to generate the sound field data in the wavenumber domain on the reconstruction surface, and finally use the spatial inverse Fourier transform to convert it to the spatial domain [7]. Similarly, this method has also been extended to the cylindrical coordinate system, forming the early cylindrical NAH theory [8].

In practice, continuous sampling is unachievable and the measuring region tends to be restricted. Therefore, for large-size measurement objects, the requirements for the number of test microphone arrays will rise sharply to ensure the coverage region and spatial sampling rate, thus increasing the test costs. To solve this problem, the patch NAH method, which can reconstruct a wide range of sound fields through a relatively small microphone array region, has emerged. Patch NAH can ensure the sampling rate of the holography measurement within a small region, while meeting the global size, data and volume requirements through interpolation and extrapolation of the measured data [9,10]. This method can realize high spatial resolution sound field reconstruction and effectively reduce the measurement cost, and is suitable for practical engineering applications. Lee and Bolton [11,12] applied the Patch NAH method to the cylindrical coordinate system, and focused on the wavenumber cut-off and parameter selection of the band-limited matrix. Based on the vanilla patch NAH method, a statistically optimal NAH (SONAH) method [12,13] has been proposed through the interpolation function and the discrete wave function value of the measurement point (small measurement region), to fit the sound field in the space basis functions to calculate the acoustic quantities on target points. Similarly, this method has also been applied to cylindrical objects [14].

On the other hand, the test costs can be reduced by measuring sparsely. There are currently two mainstream series of methods, one of which is based on the compressed sensing (CS) theory proposed by Chardon et al. [15], which can restrict the ill-posedness of underdetermined inverse problems through sparse regularization. Therefore, it can be used to solve the ill-conditioned problem of the system equation during inverse acoustical reconstruction caused by an insufficient sampling rate. Fernandez-Grande et al. combined this advantage with the equivalent source method based (ESM) NAH method [16] using the equivalent source for wave superposition, and proposed the CESM-NAH method [17]. For the equivalent sources of the CESM-NAH method, higher sparsity can lead to more accurate results. On this basis, they introduced the Laplacian operator to further enhance the sparsity of the source [18]. Based on the CESM-NAH method, Bi et al. decomposed the radiation impedance matrix into a sparse basis to enhance the accuracy of the results with a sparse equivalent source [19]. For the solution algorithm under sparse regularization constraints, Hald et al. [20] compared five algorithms and gave the corresponding selection guidance for convex optimization.

In addition, research on ensuring reconstruction accuracy when reducing the sampling rate focuses on the application of emerging deep learning (DL) theories which can be concluded as data-driven NAH methods. The domains that are currently the main centers of activity of these theories are computer vision (CV) and image processing; their application in engineering relates primarily to fault diagnosis of mechanical systems. At the core of it is the characterization of mechanical one-dimensional or processed two-dimensional signals to be recognized and learned by neural networks. In terms of fault diagnosis, the application of DL theories mainly relates to methods of autoencoder series (AE) [21], CNN series [22], recurrent neural network (RNN) series [23] and deep belief network (DBN) series [24].

The NAH also works as image processing (acoustical image) in a certain sense. For the NAH method that uses sparse measuring, the relevant issue in CV is the super-resolution of images or videos. Both the network frameworks capable of image super-resolution [25] built through deep CNN and those capable of video super-resolution [26] built through 3D-CNN have achieved impressive results. At the same time, the framework of the generative adversarial networks (GAN) stacked with CNN layers has achieved an effect close to that achieved by supervised learning in terms of unsupervised image super-resolution and denoising [27]. Therefore, these DL theories have the potential to be applied to sparsely sampled NAH methods including NAH methods based on Bayesian learning [28] or Bayesian inference [29,30], the method based on a generative model [31] to realize near-field acoustic reconstruction for large sources, and a series of methods based on 2D-CNN. These methods include the KHCNN-NAH method proposed by Olivieri [32,33] et al. based on 2D-CNN, the Kirchhoff–Helmholtz integral formula and the vibration mode recognition method based on 2D-CNN [34] proposed by Wu et al. They have a high level of accuracy in restoring the shape of a single mode shape. The CSA-NAH [35] and its improved jointly trained CSA-NAH [36] method based on 3D-CNN and the stacked autoencoder (SAE) method proposed by Wang et al. process the acoustical image including the frequency spectrum, which can make use of the frequency dimension information to a certain extent, thus retaining the reconstructed spectral information.

For the relevant NAH methods that adopt CS theory, highly random sampling is required [19] in the application process. At the same time, it is essential to select the appropriate base or dictionary for certain applied issues, otherwise the accuracy of the results will be affected [37]. In addition, the solution algorithm for the 1-norm constraint problem of sparse regularization is usually stable but slow [20], so there are some limitations in application. For the CS-based NAH method, there is a comparison of reconstruction accuracy in [35], which shows that the error of the 3D-CNN based method is lower. For common 2D-CNN-based NAH methods, as a result of the lack of frequency information or each group of graphs having been normalized separately, which accounts for the spectral information lost, the frequency information is not effectively utilized either. This is illustrated in [36] and in the accuracy comparison. For Bayesian-related methods, it is necessary to assume an appropriate prior distribution. Therefore, we choose the 3D-CNN-based CSA-NAH method over others. 

In terms of the CSA-NAH method, the research object is the planar structure; the 3D-CNN layer can effectively act on the planar three-dimensional acoustical image. Based on the chosen method, we introduce a feasible cylindrical truncation and extension scheme, so that the 3D-CNN layer can convolute with cylindrical three-dimensional images, thus realizing the cylindrical NAH under sparse sampling. The method is called CS3C-NAH, and adopting this method can significantly reduce the measuring sampling rate while ensuring sufficient reconstruction accuracy. This lowers the industrial application threshold of the NAH method, improving stability, and reducing financial costs of implementation. The main work of this paper is as follows:

- Briefly state the theory of cylindrical NAH, and give the reason for the error in the results when the holographic sampling rate is reduced;
- Propose the CS3C-NAH model combining SAE, 3D-CNN and a feasible truncation and padding method;
- The patch NAH method using the Papoulis–Gerchberg extrapolation interpolation algorithm (PGa) is introduced into the cylindrical coordinate system to realize cylindrical NAH;
- The CS3C-NAH method is numerically verified, and compared with the patch NAH method based on cylindrical PGa to verify the feasibility of the proposed method.

## 2. Theory of the Spatial Fourier Transform-Based Cylindrical NAH and Aliasing

### 2.1. Theory of SFT-CNAH

**Figure 1:**
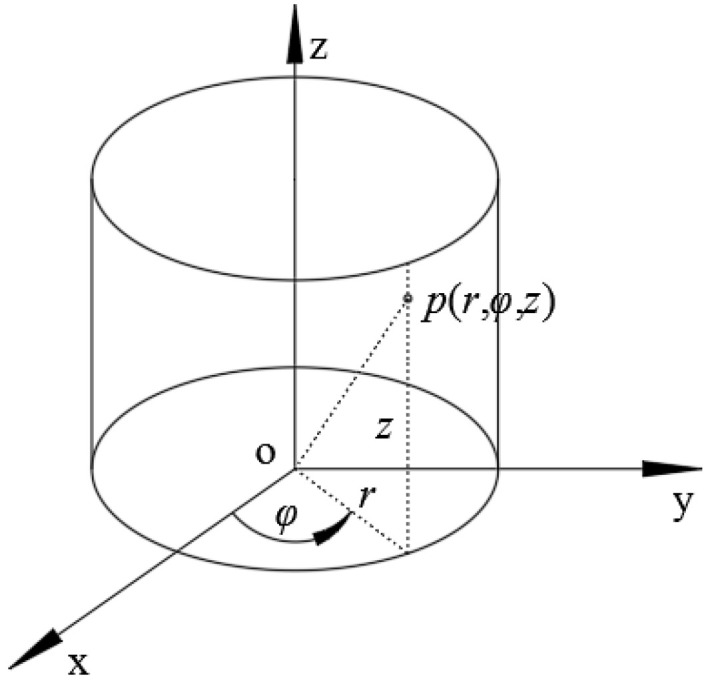
The spatial distribution of sound pressure in the cylindrical coordinate system.

The spatial distribution of the complex sound pressure in the cylindrical coordinate system is shown in Figure 1. The source, holographic (or measurement surface) and reconstructed surface, are all outside the sound source. This paper focuses on reconstructing the sound pressure on the reconstructed cylindrical surface based on sparsely measured pressure.

Suppose the cylindrical holography and reconstruction surfaces surrounding the analysis domain are labeled as *C_h_* and *C_c_* with the radius *r_h_* and *r_c_*, respectively. The pressure distributions on them are *p*(*r_h_*_,_ *φ, z*) and *p*(*r_c_*_,_ *φ, z*). Traverse *φ, z* to perform 2D spatial Fourier transform on *p*(*r*_,_ *φ, z*). Due to the fact that the range of *φ* in cylindrical coordinate system is 0~2π, it can be periodically extended. Thus, the spatial Fourier transform of parameters *φ* in fact solves the Fourier series, and the helical wave spectrum of the sound pressure *P_n_*(*r*,*k_z_*) can be obtained:

(1) $$P_{n}\left(r_{h},k_{z}\right)\equiv F_{\varphi }F_{z}\left[p\left(r_{h},\varphi ,z\right)\right]=\frac{1}{2\pi }\int_{0}^{2\pi }e^{-in\varphi }d\varphi \int_{-\infty }^{\infty }p\left(r_{h},\varphi ,z\right)e^{-ik_{z}z}dz$$

The subscript n in Equation (1) is the circumferential component serial number of the cylindrical wave, and *F_φ_F_z_* represents the Fourier series of the variable *φ* and *z*. For the exterior acoustic problem, the cylindrical wave spectrum on *C_c_* can be obtained by the extrapolation of the helical wave spectrum on *C_h_*:

(2) $$P_{n}\left(r_{c},k_{z}\right)=\frac{H_{n}^{\left(1\right)}\left(k_{r}r_{c}\right)}{H_{n}^{\left(1\right)}\left(k_{r}r_{h}\right)}P_{n}\left(r_{h},k_{z}\right)$$

where *k_r_* is radial wave number:

(3) $$\left\{\begin{array}{c} k_{r}=\sqrt{k^{2}-k_{z}^{2}},\;k_{z}^{2}\leq k^{2}\\ k_{r}=i\sqrt{k_{z}^{2}-k^{2}},\;k_{z}^{2}>k^{2} \end{array}\right.$$

and where *k* denotes the wave number, *k* = *ω*/*c*. *ω* is the angular frequency and *c* is the sound propagation velocity in the medium. $H_{n}^{\left(1\right)}$ refers to *n*th-order Hankel function of category I and corresponds to the wave of outward divergence.

(4) $$H_{n}^{\left(1\right)}\left(k_{r}r\right)=J_{n}\left(k_{r}r\right)+iY_{n}\left(k_{r}r\right)$$

*J_n_* and *Y_n_* represent category I and II Bessel function, respectively. Perform inverse spatial Fourier transformation on *P_n_* (*r_c_*, *k_z_*) to derive the sound pressure on *C_c_*.

(5) $$p\left(r_{c},\varphi ,z\right)=F_{\varphi }^{-1}F_{z}^{-1}\left[P_{n}\left(r_{c},k_{z}\right)\right]=\overset{\infty }{\underset{n=-\infty }{\sum }}e^{in\varphi }\frac{1}{2\pi }\int_{-\infty }^{\infty }P_{n}\left(r_{c},k_{z}\right)e^{ik_{z}z}dk_{z}$$

The implementation of SFT-based cylindrical NAH is derived from the combination of Equations (2) and (5):

(6) $$p\left(r_{c},\varphi ,z\right)=\overset{\infty }{\underset{n=-\infty }{\sum }}e^{in\varphi }\frac{1}{2\pi }\int_{-\infty }^{\infty }\frac{H_{n}^{\left(1\right)}\left(k_{r}r\right)}{H_{n}^{\left(1\right)}\left(k_{r}r_{h}\right)}P_{n}\left(r_{h},k_{z}\right)e^{ik_{z}z}dk_{z}$$

**Figure 2:**
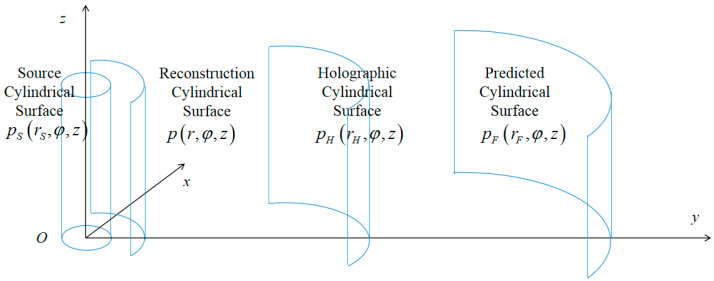
Cylindrical wave near-field reconstruction and far-field prediction.

According to Equation (6), the principle of cylindrical NAH is shown in Figure 2.

### 2.2. Aliasing Caused by Sparse Sampling

It is to be noted that SFT-CNAH cannot achieve continuous sampling during the application process. Due to the periodicity of the circumferential sound field, the helical wave spectrum is discrete in the circumferential wave number, so aliasing in the *z* axial wave number must take place.

The essence of limited region discrete sampling is to perform windowing and sampling processing on a continuous spectrum as:

(7) $$p\left(r_{c},\varphi ,z\right)=F_{z}^{-1}\left\{\frac{H_{n}^{\left(1\right)}\left(k_{r}r_{c}\right)}{H_{n}^{\left(1\right)}\left(k_{r}r_{h}\right)}F_{z}\left[p\left(r_{h},\varphi ,z\right)\right]\cdot \Pi \cdot S\right\}$$

The rectangular window function *Π* acts as a window effect, while sampling function *S* the spatial discreteness:

(8) $$S\left(\frac{z}{\Delta_{z}}\right)=\left|\Delta_{z}\right|\underset{m}{\sum }\delta \left(z-m\Delta_{z}\right)$$

Δ*z* is the sampling interval. To facilitate the analysis of the aliasing, the window effect and wavenumber discreteness are ignored. Equation (7) can be simplified to:

(9) $$p\left(r_{c},\varphi ,z\right)=F_{z}^{-1}\left[\frac{H_{n}^{\left(1\right)}\left(k_{r}r_{c}\right)}{H_{n}^{\left(1\right)}\left(k_{r}r_{h}\right)}\underset{m}{\sum }P_{n}\left(r_{h},k_{z}-\frac{2\pi }{\Delta z/m}\right)\right]$$

After the discrete sampling, the helical wave spectrum of the measured sound pressure will be composed of the *p*(*r_h_*, *φ*, *z*) within the measurement aperture and the adjacent virtual images distributed by the periodic replication and extension.

It can be seen from Equation (9) that, if the concerned upper limit of the band-limited wavenumber *k_z_* is *k_zm_*, then when the sampling interval Δ*z* cannot satisfy Δ*z* < 2π*m*/*k_zm_*, aliasing will occur at the boundary of the helical wave spectrum, and the severity of aliasing increases with the increase in sampling interval, resulting in poor performance in reconstruction.

## 3. Methodology of the CS3C-NAH

**Figure 3:**
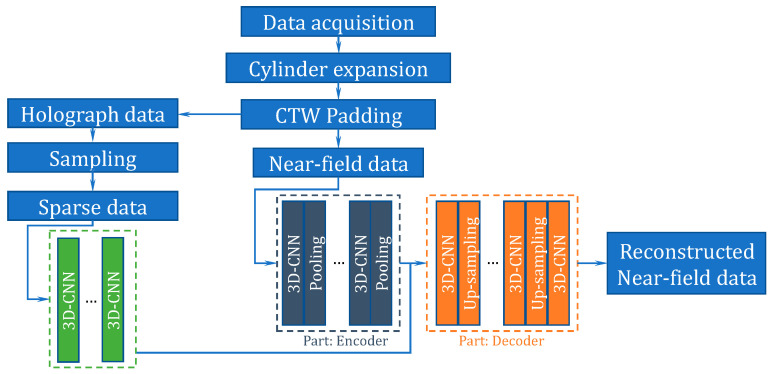
Implementation process of the CS3C-NAH method.

To reduce the impact on the accuracy of the reconstruction resulting from aliasing caused by discrete sampling, this paper proposes a method used for recovering the acoustical data from sparse to dense which is based on SAE and 3D-CNN and is commonly used in the fields of video classification and action recognition by extracting and learning the graphic and time-sequential information of the video. The frequency spectrum of cylindrical sound pressure distribution *p*(*r*, *φ*, *z*, *f*) in CNAH is essentially 4D images. When the radius *r* is specified, and the cylindrical surface is truncated and unfolded into planar, the sound pressure distribution spectrum to be processed is transferred to 3D images, and the spatial coordinates are (*φ*, *z*, *f*). Unlike the traditional planar CNN, the cylindrical CNN uses a cylindrical translation window (CTW) first, so that the original circumferential and continuous characteristics at the truncated edge can be appropriately supplemented and extracted. Based on CTW, SAE and 3D-CNN, a new method named CS3C-NAH is proposed. The specific implementation process of the CS3C-NAH method is shown in Figure 3.

The method is mainly divided into three parts. First is the acquisition and processing of data. Next, the near-field data are selected to train the SAE composed of an encoder and a decoder to optimize the framework and hyperparameters. Finally, several 3D-CNN layers are stacked for feature extraction and integrated into the front end of the optimized decoder for final joint training. The well-trained network can reconstruct the dense near-field acoustic quantities by inputting sparse holographic data into it.

### 3.1. Cylindrical 3D-CNN with CTW

The deep CNN model can integrate features within a large spatial range for the original input images after translation, rotation or scaling operations, and has stable spatial invariance facing with the small changes of the feature position by pooling layers. Although the convolutional layer is not spatially invariant, it builds high-level features by combining low-level features. After a deep hierarchy of the convolutional and pooling layers, the CNN model can capture more complex features. Thus, the last pooling layer captures the highest level of features, and has the strongest spatial invariance between the convolutional and pooling layers.

**Figure 4:**
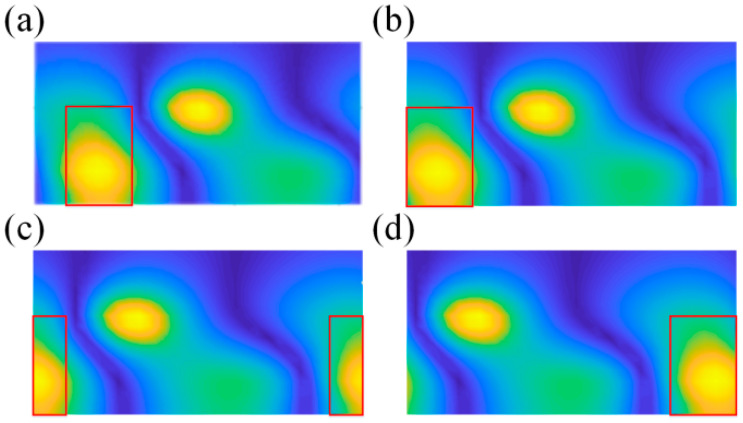
Truncated and unfolded three-dimensional data for feature extraction. (**a**) is the original rolled out image, (**b**–**d**) image by moving the original image by Δ*px*_1_, Δ*px*_2_ and Δ*px*_3_ pixels to the left edge. Red rectangle is the receptive field in input image mapped by fixed pixels in a pooling layer.

**Figure 5:**
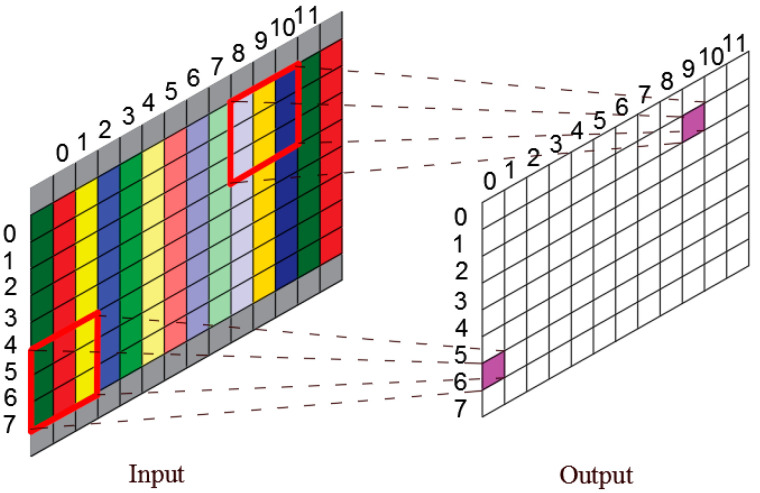
Operation mechanism of the cylindrical translation window. The green pixels in the left-most column and red pixels in the right-most column are duplications from the opposite edges.

Ignoring the frequency dimension, the 2D planar data *p*(*φ*, *z*) obtained from the truncation of spatial cylindrical data *p* is the input data of CCNN. Figure 4a is the original rolled out image, and Figure 4b–d are created by moving the original image by Δ*p_x_*_1_, Δ*p_x_*_2_ and Δ*p_x_*_3_ pixels to the left edge. The red rectangle is the receptive field in input image mapped by fixed pixels in a pooling layer. When the CNN structure is trained with the images shown in Figure 4, it can capture and learn important features of the sound pressure distribution because of the existence of images translated to a different location (Figure 4a,b,d) on the basis of the translation invariance of the pooling layer. However, when the new image (Figure 4c), which originates from moving the original image by Δ*p_x_*_2_, is taken as the input data, the CNN model may not identify the sound source because the image feature is split up and the relative position is switched. To solve this problem, this paper combines CWT and CNN to form cylindrical CNN. The operation mechanism of CTW is shown in Figure 5.

To avoid performing zero padding at the left and right boundaries of the image which will be inputted to the CNN layer, units in the first column (column 0) on the left are copied to the boundary on the right, and units in the last column (column 11) on the right are copied to the boundary on the left. Zero padding continues to be applied to the top and bottom boundaries. In this way, the truncated and rolled-out planar graph can be transformed into an equivalent cylindrical graph. Convolve it with 3D-CNN to realize feature extraction and perception. CTW horizontally expands the size of the receptive field of the unit at the truncated boundary of the original input image while the frequency dimension remains unchanged. By modifying the input images with CTW, the cylindrical 3D-CNN layers are formed together with 2D pooling layers and activation functions. 

**Figure 6:**
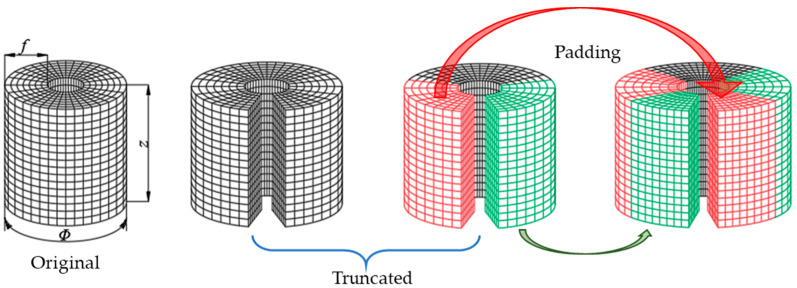
Detailed illustration of processing the cylindrical data using the CTW.

As shown in Figure 6, the original near-field sound pressure data that radiated from a set of dipole source pairs is continuously distributing in the radian range 0~2π, in which the truncation will be performed at a certain radian. The receptive field corresponding to the boundary unit contains less information than that which corresponds to the center unit. To ensure that each unit of convoluted maps maintains the same receptive field size as the original image, the image boundary data are padded with switched units marked in red and green, horizontally. The left column is padded with green units on the right side and the other column is padded with green units on the left. It can be seen that the cylindrical 3D-CNN can process the input image with CTW to expand the boundary units’ receptive field without adding new data. Although the receptive field of modified layer boundary units still contains less information than that of central units, the information contained in boundary units’ receptive field is supplemented to a certain extent.

**Figure 7:**
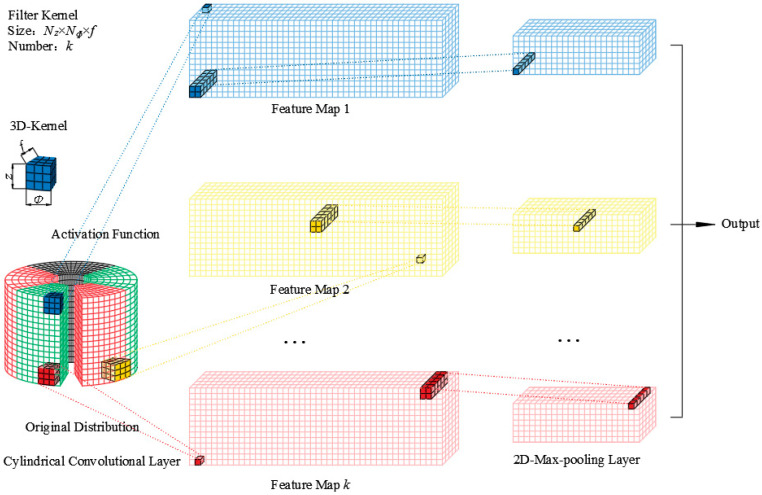
The principle of the cylindrical 3D-CNN.

The processed 3D acoustic images are convolved with the kernels of 3D-CNN. The convolution process is shown in Figure 7.

Several 3D convolution kernels are convoluted with the processed images in the specified direction (*z*, *φ*, *f*). The output of the convolution operation is as follows:

(10) $$N_{xyi}=\varphi \left(R_{k}\cdot M_{xyi}+b\right)$$

where ***R**_k_* denotes the tensor corresponding to the *k*-th convolution kernel, and ***M**_xyi_* is the 3-order tensor of input data with the same size as ***R****_k_* starting from (*x*, *y*, *i*) in *z*, *φ* and *f* directions. Point multiplication ‘·’ represents the inner product of two tensors. *b* is the bias parameter after the convolution operation. *φ*(*) is the activation function in Figure 7. The common activation functions are: Sigmoid, ReLU, Tanh, Leaky ReLU, etc.

### 3.2. Cylindrical 3D-CNN SAE Module

**Figure 8:**
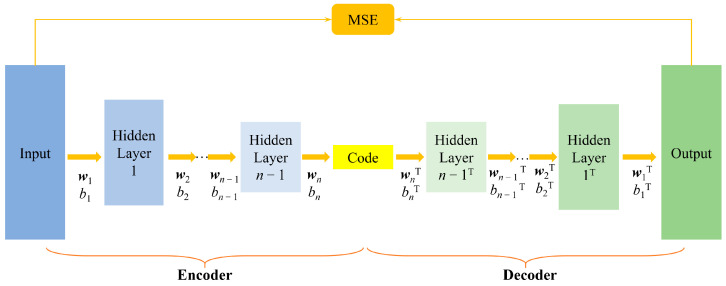
The principle of the SAE. {$w_{n}$, $b_{n}$} and {$w_{n}^{T}$, $b_{n}^{T}$ are the weight and bias parameters of the n-th encoder and decoder, respectively (superscript T does not denote transposition).

A pooling layer is used to prioritize features and extract relatively important ones. The 2D pooling layer connected after the image extraction nodes can be used to compress the data and capture its hierarchy information. The pooling operation first divides the input images into several rectangular regions. The mean pooling layer is then used to average the image pixels in the regions, or the max pooling layer is used to maximize the value. Of the obtained features, the former is more sensitive to the background information while the latter texture has greater feature information. The up-sampling procedure is the inverse of pooling, and is commonly based on an appropriate method of internal interpolation. In this paper, max pooling and up-sampling based on bilinear interpolation are adopted. Firstly, the compressed characteristics of the theoretical sound pressure data on the reconstructed surface are obtained through 3D-CNN and max pooling. The images will then be restored based on compressed features via 3D-CNN and up-sampling. This will constitute the SAE structure as shown in Figure 8.

**Figure 9:**
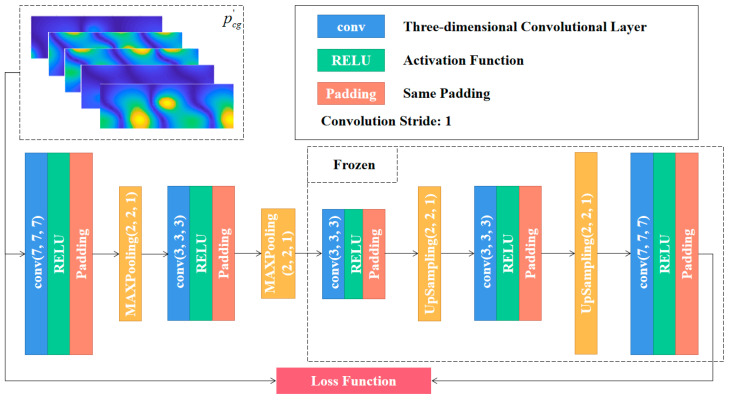
The SAE used to handle theoretical sound pressure on the reconstructed surface. $p_{cg}^{\prime }$ represents theoretical sound pressure of the reconstructed surface after the CTW processing. The ‘conv’ represents the 3D convolution layer, the three dimensions correspond to the axial (*z*), circumferential (*φ*) and the frequency dimension (*f*), respectively. The two dimensions of max pooling and up-sampling correspond to the axial (*z*) and circumferential dimension (*φ*), respectively. All convolutional layers use ReLU (max (0, *x*)) as the activation function. The stride is 1 and ‘same padding’ is used.

The mean square error (MSE) of the theoretical and the decoded sound pressure data is selected as the loss function during training of the SAE network. The data above are all processed by CTW. As shown in Figure 8, the parameters will be determined by minimizing the loss function *L*.

(11) $$L=\frac{1}{W}{\left\|{p^{\prime }}_{cg}\left(s_{t},z_{x},\varphi_{y},f_{i}\right)-{\overset{¯}{p}}_{cg}\left(s_{t},z_{x},\varphi_{y},f_{i}\right)\right\|}_{F}$$

where ${\left\|*\right\|}_{F}$ denotes the Frobenius normalization, *s_t_* is the serial number of the excitation, and *W* is the number of data sets. The SAE used to handle theoretical sound pressure on the reconstructed surface is shown in Figure 9. It should be noted that, to avoid repeated descriptions in the following parts, the hyperparameters of the network structure given here are the optimization results explored in the subsequent numerical calculation and validation section, and can be referred to as default values. However, the specific hyperparameters should be explored based on [36] to achieve better performance during practical application. The encoder in the figure consists of two 3D-CNN layers (the kernel numbers are all 8) and two max pooling layers for simulation of 1/4 × 1/4 sparse sampling. The feature extraction and compression of the data on the reconstructed surface is performed to realize the sparse sampling of the sound pressure on the holography surface. The decoder in Figure 9 consists of 3 3D-CNN layers (the kernel numbers of the first two are both 8, and the last one is 1) and two up-sampling layers (the size of the output data is 4 × 4 times that of input). The size of the hidden-layer data is magnified and recovered to the scale of the original sound pressure on the reconstructed surface.

The number of layers in the SAE can be increased or decreased in practice: multiple stacks can perform further compression of the data, while producing more general and beneficial features. The accuracy of the reconstructed image will be greatly affected if the hyperparameters are selected poorly.

Gradient descent with error propagating backward is used to train the network by minimizing the loss function of Equation (11). The weights and bias of all networks form the parameter set *β* with the initial value *β*_0_. The gradients of the loss function L for all parameters are solved in each iteration. On the basis of the gradient, the parameters set *β* can be iterated as follows: $\beta^{n+1}=\beta^{n}-\eta \nabla L\left(\beta^{n}\right)$. Until $\nabla L\left(\beta^{n}\right)\approx 0$, the iteration is terminated. *η* is the learning rate. In the case where the gradient fluctuates significantly, the constant learning rate will lead to the failure in convergence. The optimizer entitled Nadam (Nesterov-accelerated adaptive moment estimation), which has an adaptive learning rate, is utilized here to update the parameter set. In order to improve the efficiency of training, the data are divided into batches, and the batch size can be increased moderately to accelerate the convergence under the limitation of computer capability. The samples are divided into a training set and a test set to prevent overfitting in training. Train the network until the validation loss converges.

The training of neural networks is a forceful attempt to find an optimal prediction model through the training set. During parameter iteration and error convergence, the weight and bias are determined. The trained model is then used for the prediction of the same object. The pre-training process of the SAE structure can independently optimize the hyperparameters of the decoder with the function of enlarging the data size, so that the number of hyperparameter groups to be compared and optimized is greatly reduced from the multiplier level to the addend level, thus reducing the workload for model adjustment and optimization [36].

### 3.3. Combined CS3C Model

**Figure 10:**
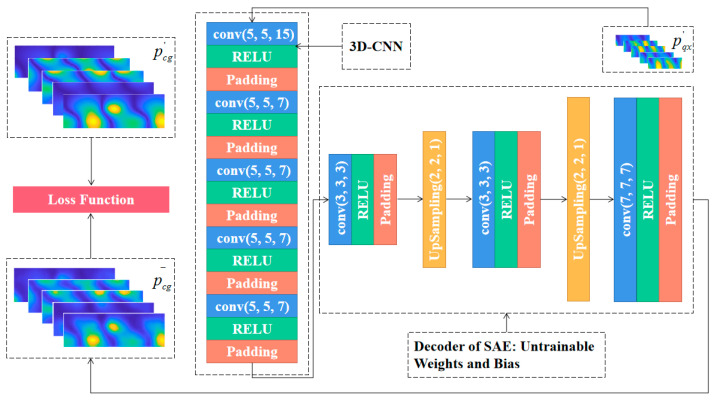
The flow of the CS3C-NAH model.$p_{qx}^{\prime }$ and $p_{cg}^{\prime }$ represent sound pressure data after CTW processing on the reconstructed surface and the hologram surface, respectively. ${\bar{p}}_{cg}$ represents sound pressure rebuilt by the CS3C-NAH model. The loss function, activation function and the optimizer are the same as the SAE in the previous section. The stride is 1 and ‘same padding‘ is used.

In order to reconstruct the dense sound pressure data on the reconstructed surface using the sparse sound pressure data on the hologram surface, the model containing CTW, SAE and 3D-CNN is established. The model is named CS3C and is shown in Figure 10. Firstly, the SAE is disassembled, and the parameter sets of well-trained decoders are fixed to be untrainable in subsequent operations. Then the stacked 3D-CNN layers without pooling and up-sampling are used to extract the features of sparse sound pressure data on the hologram surface processed by CTW. Finally, the output feature maps will be put into a fixed decoder. To summarize, the combined CS3C model is composed of stacked 3D-CNN layers for feature extraction and a decoder for data expansion. The loss function is the MSE between the final output of the decoder and the theoretical sound pressure data on the reconstructed surface instead of being the MSE between the output of the stacked 3D-CNN layers and the hidden layers of SAE. This reduces the influence of the inherent error of the decoder. 

The flow of the CS3C-NAH model for reconstructing the dense sound pressure data on the reconstructed surface based on the sparse sound pressure data on the hologram surface is shown in Figure 10. The stacked layers for feature extraction are 5 3D-CNN layers (the kernel number of the first one is 16, and the last four are all 8). The features of sparse sound pressure data on the hologram surface are extracted to obtain the hidden layer containing the compressed features. The decoder in Figure 10 is the same as that of SAE. The hidden-layer data are expanded and recovered to the scale of the original sound pressure on the reconstructed surface.

Finally, the trained CS3C-NAH model can retain enough information to reconstruct the sound field after reducing the spatial sampling rate.

## 4. Numerical Example and Comparison

### 4.1. Acquisition of Data

A model of a series of dipole source pairs in an air medium are simulated using COMSOL. The position range of point source 1 in cylindrical coordinates is (0~0.057 m, 0~2π rad, 0~0.160 m), while point source 2 is (0~0.057 m, 0~2π rad, 0.160~0.320 m). The bottom surface is a rigid sound field boundary with *z* = 0. The details of the simulation are as follows: radius of holographic cylindrical surface, *r_h_* = 0.1415 m; radius of reconstructed cylindrical surface, *r* = 0.09 m; upper bound radius of source cylindrical surface radius *r_s_* = 0.057 m; the sound of speed *c* = 343 m/s; and the air density *ρ* = 1.29 kg/m^3^.

The absolute value of the complex sound pressure data on the hologram surface is obtained from sparse sampling and named *p_qx_*. The absolute value of the theoretical sound pressure data on the reconstructed surface named *p_cg_* is obtained from dense sampling. *p_qx_* and *p_cg_* are both obtained through the joint simulation developed using COMSOL and MATLAB.

There are 1000 dipole source pairs with different position and excitation value. The excitation intensities of the two sources are both between 0 W and 0.0001 W. The simulation is performed at 96 frequency points (100~2000 Hz; the interval is 20 Hz). The SNR in the simulation is 25 dB.

For *p_cg_*, the measuring points are uniform distributed along circumferential and axial directions with the sampling number *N_φ_* = 32 and *N_z_* = 16. The microphone spacing in the circumferential direction and the axial direction is Δ*φ* = 0.196 rad and Δ*z* = 0.02 m, respectively. The size of the sampled data tensor is (none, 16, 32, 96). As for *p_qx_*, the measuring points are uniform distributed along circumferential and axial directions with the sampling number *N_φ_* = 8 and *N_z_* = 4. The microphone spacing in the circumferential direction and the axial direction is Δ*φ* = 0.785 rad and Δ*z* = 0.08 m, respectively. The size of the sampled data tensor is (none, 4, 8, 96). Finally, apply CTW to *p_qx_* and *p_cg_*, respectively. The pixel width of the former’s padding unit is 8, and that of the latter is 2. The sizes of $p_{qx}^{\prime }$ and $p_{cg}^{\prime }$ used for network training are (none, 16, 48, 96) and (none, 4, 12, 96), respectively.

### 4.2. Normalization

The amplitude of the raw spectrum at the low frequencies far outweighs that observed at high frequencies. At the same time, the source strength of the different dipole source pairs in the spatial domain is also distinguished. The difference of sound pressure amplitude among all frequencies and samples is too large, which will cause the loss function to assign excessive weights to the low frequencies and samples with large source strength. The domain points with a small amplitude will be directly ignored, so that the information from all domain points cannot be considered globally, which would result in inconvergence or poor performance of the networks. Meanwhile, the fluctuations of some signals are too small to be sufficiently perceived by the convolution kernels of 3D-CNN.

To eliminate the impact of wide-ranging amplitude distribution in the frequency and spatial domain on neural networks, a data normalization method for CS3C-NAH is introduced as in [35]. While retaining the ability of feature extraction for the signal with low frequencies and large source strength, more weight is allocated to the component at medium and high frequencies and samples with small source strength. The core of the normalization method is to traverse all data along space and sample dimensions, and scale the value to (0,1) at each frequency point. This is because the sound pressure on the reconstructed and the holography surface maintains a linear mapping relationship. Therefore, the sound pressure data on the latter should also be normalized with the identical scaling factors via the same method.

**Figure 11:**
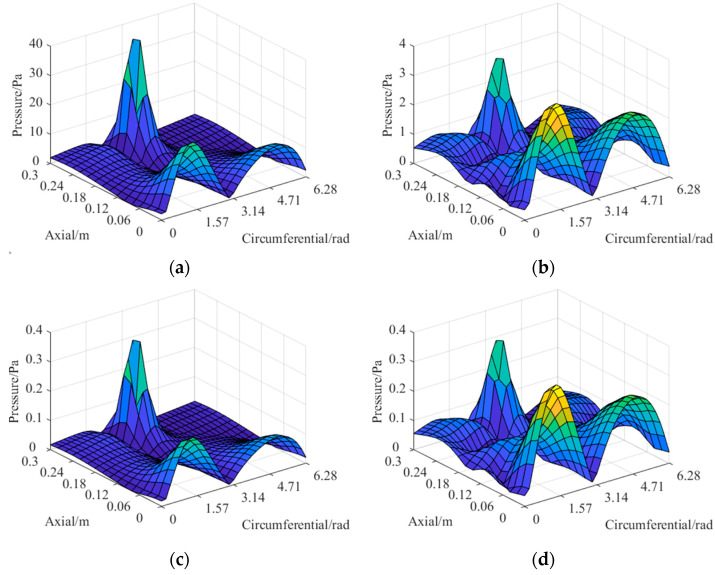
The normalization of sound pressure data on the reconstructed surface: (**a**) raw data at 100 Hz; (**b**) raw data at 2000 Hz; (**c**) processed data at 100 Hz; (**d**) processed data at 2000 Hz.

The theoretical sound pressure on the reconstructed surface and the sparse sound pressure on the holographic surface should be normalized as described above before being used to train the network. After the generalization of the CS3C-NAH model, the factors of data normalization will be used to restore the reconstructed sound pressure on the reconstructed surface reversely. Selection of a dipole point source pair is used to illustrate the effect of normalization. One dipole point source’s excitation value is 8.2E-5 W at (*r* = 0.0146 m, *φ* = 0.89 rad, *z* = 0.001 m). The other 4.3E-5 W at (*r* = 0.0556 m, *φ* = 2.74 rad, *z* = 0.246 m). The compared results for the normalization effect are shown in Figure 11.

It can be seen from the comparison that the amplitude of the processed data is limited to the range (0, 1), which greatly reduces the amplitude difference in the original data and effectively enhances the convergence of the neural network.

### 4.3. Data Processing by CTW

**Figure 12:**
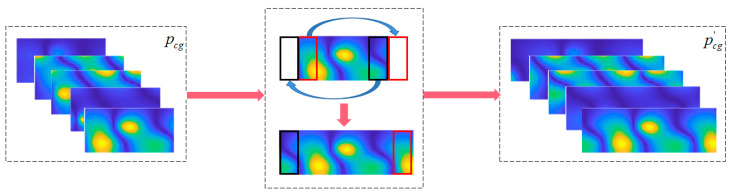
The horizontal translation and padding of the left and right boundary of the data on the reconstruction surface (CTW).

CTW is used to process the normalized theoretical sound pressure of the reconstructed surface and the sparse data of the measuring surface. The process is illustrated in Section 3.1. For a certain sample and frequency point of data on the reconstruction surface, the pixel width of the padding unit is 8. The size of reconstructed surface sound pressure data will be padded from the size of 16 × 32 to 16 × 48 ($p_{qx}^{\prime }$), as shown in Figure 12.

**Figure 13:**
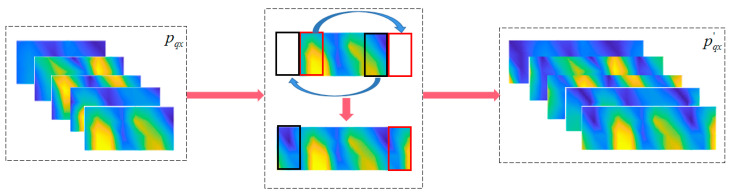
The horizontal translation and padding of the left and right boundary of the sparse data on the measuring surface (CTW).

For a certain sample and frequency point of sparse data on the measuring surface, the pixel width of the padding unit is 2. The size of holography surface sound pressure data will be padded from the size of 4 × 8 to 4 × 12 ($p_{cg}^{\prime }$), as shown in Figure 13.

### 4.4. Network Training and Results

All the programs used for calculation of neural networks are compiled based on the available platforms: TensorFlow2.1.0, Keras 2.3.1 and Python 3.6. The hardware environment used for compiling consists of the CPU (Intel Xeon E5 @ 2.60GHz), RAM (256GB), GPU (NVIDIA Tesla K20c) and OS (Windows 10).

**Figure 14:**
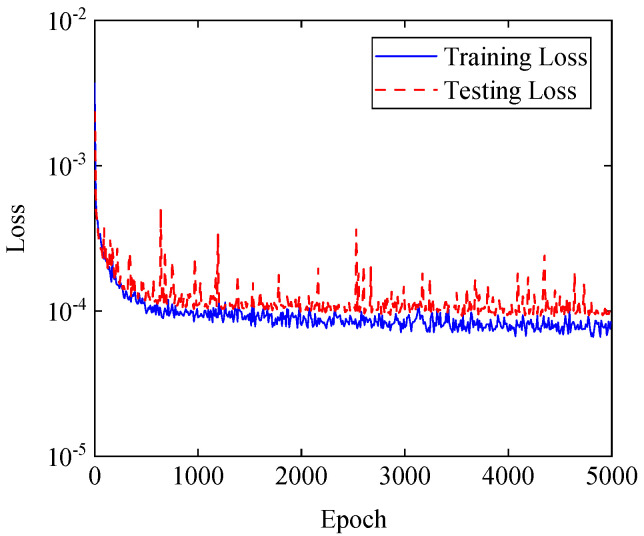
Training and testing loss of CS3C−NAH.

The sample size of data sets composed of the sound pressure data on the reconstructed and holographic surface processed by CTW is 1000 in terms of different excitation points. Thus, the sizes of data on reconstructed and holographic surfaces are (1000, 16, 48, 96) and (1000, 4, 12, 96), respectively. The 1000 data samples will be divided into training and test sets at a ratio of 8:2. The batch size for training is 16, and the training epoch size is 5000. After the SAE model has been trained sufficiently, the training and testing loss of the CS3C-NAH model are shown in Figure 14.

In Figure 14, the training loss is close to the testing loss, and the model converges with slight fluctuation. Finally, the training loss is 1.04 × 10^−4^ and the testing loss is 1.24 × 10^−4^. The reason why the testing loss fluctuates at the late stage of the iteration is that the batch is small. The fluctuation can be reduced by enlarging batch size appropriately, but the average testing loss will increase.

#### 4.4.1. Reconstruction Error

The CS3C-NAH model is trained using the theoretical sound pressure data (1000 × 16 × 48 × 96) and the sparse sound pressure data (1000 × 4 × 12 × 96) after CTW treatment. Next, the reconstructed sound pressure is obtained on the reconstructed surface by generalization. To assess the error of reconstructed sound pressure data compared to the theoretical at each frequency point, use the Equation (12) to calculate error.

(12) $$e=\frac{\sqrt{\sum_{x,y}{\left(p_{cg}^{\prime }\left(z_{x},\varphi_{y}\right)-\left(z_{x},\varphi_{y}\right)\right)}^{2}}}{\sqrt{\sum_{x,y}p_{cg}^{\prime }{\left(z_{x},\varphi_{y}\right)}^{2}}}$$

A certain dipole source pair is chosen to explore the reconstruction error frequency (100~2000 Hz) spectrum of the CS3C-NAH method. The intensity and position (*r*, *φ*, *z*) of the source pair are 7.97 × 10^−5^ W, 1.94 × 10^−5^ W and (0.01253 m, 1.44 rad, 0.0335 m), (0.0442 m, 3.85 rad, 0.182 m), respectively.

**Figure 15:**
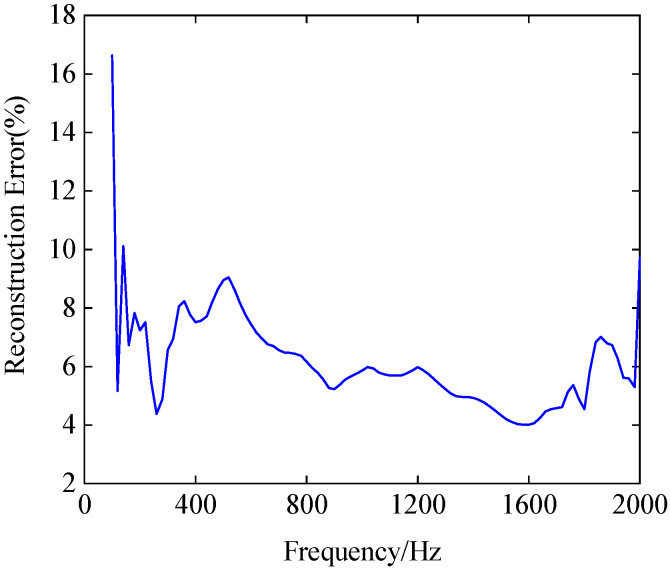
The reconstruction error of the CS3C-NAH method.

In Figure 15, the overall error is below 10%, except for at 100 Hz and 120 Hz, where the errors are 16.63% and 10.1%, respectively. The average error is 6.14%. After excluding points exceeding 10% at 100 Hz and 140 Hz, the maximum and average error are 9.69% and 5.99%, respectively. In general, the CS3C-NAH method can achieve a high level of accuracy in reconstructing acoustic quantities at high frequencies under the premise of sparse sampling.

To verify the sound field’s reconstruction accuracy using the CS3C-NAH model for different dipole source pairs, 10 groups of dipole source pairs are selected and the performance at 200 Hz is chosen for exploration. The position and excitation intensity of the dipole source pairs are listed in Table 1.

**Figure 16:**
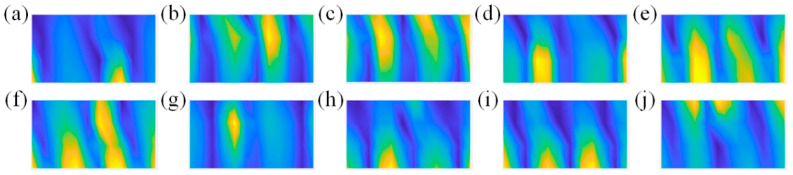
Amplitude distribution of the sparse sound pressure on the hologram surface. (**a**–**j**) represent different dipole source pairs.

The sound pressure distributions excited by the dipole source pairs given above are sparsely sampled on the hologram surface. Their amplitude distributions are shown in Figure 16.

**Figure 17:**
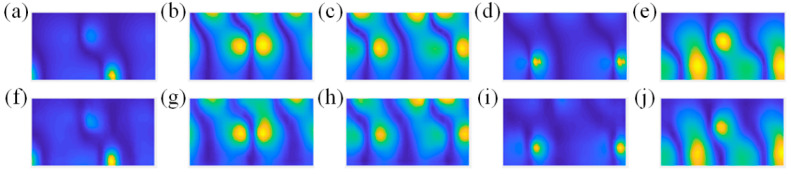
The comparisons of the theoretical and reconstructed sound pressure distributions on the reconstructed surface of the former five source pairs in Figure 16. (**a**–**e**) represent the theoretical sound pressure of the former five source pairs in Figure 16. (**f**–**j**) represent the corresponding reconstructed sound pressure.

**Figure 18:**
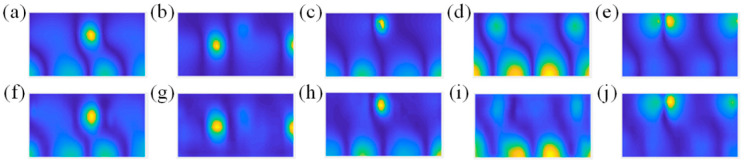
The comparisons of the theoretical and reconstructed sound pressure distributions on the reconstructed surface of the latter five source pairs in Figure 16. (**a**–**e**) represent the theoretical sound pressure. (**f**–**j**) represent the corresponding reconstructed sound pressure.

The comparisons of the theoretical and reconstructed sound pressure distributions are shown in Figure 17 and Figure 18. The errors of the former five source pairs calculated by Equation (11) are shown in Table 2. The errors of the latter are shown in Table 3.

According to Figure 17 and Figure 18, the method can accurately locate the spatial position of the sound source from the perspective of standing waves. Meanwhile, it can be seen from the specific error in Table 2 and Table 3 that the CS3C-NAH method can accurately reconstruct not only the shape of the relative distribution of sound pressure amplitude, but also the absolute values at each point globally. The average reconstruction error of the 10 source pairs at 200 Hz is only 9.6% lower than 10%.

#### 4.4.2. Comparison of the Methods

It is desirable to verify whether the proposed method improves the sound field reconstruction accuracy when compared to other methods for the same sampling number. The PGa-based patch NAH method is selected to be compared under the same conditions as the CS3C-NAH method. 

The position and excitation intensity of the dipole source pairs for comparison are listed in Table 4. Before the methods are applied, the data normalization is utilized for the sound pressure data, and then CTW is performed. The pressure data *p_cg_*, *p_qx_* and $p_{cg}^{\prime }$, $p_{qx}^{\prime }$ after CTW processing are of the same size as those of the numerical example above. In the axial dimension, the array covers from 0 to 0.320 m, and the SNR for sparse pressure on the measuring surface is 20 dB.

**Figure 19:**
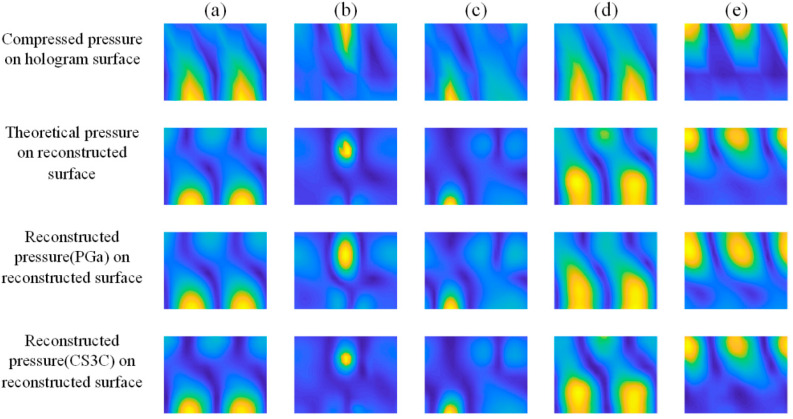
The relative amplitude distributions of sound pressure distribution on the hologram and reconstructed surface. (**a**–**e**) correspond to five dipole source pairs. PGa and CS3C are the abbreviations of the PGa-based Patch NAH and CS3C-NAH methods, respectively.

The theoretical sound pressure data on the holographic and reconstructed surface for the dipole source pairs shown in Table 4 are calculated, and the former is 1/4 × 1/4 sparse sampled to obtain the data *p_cg_*, *p_qx_* respectively for comparison. Finally, all the data are applied to the CS3C-NAH method and the PGa-based Patch NAH method, and the reconstructed sound pressure data of the two methods, respectively, are obtained. The error between the reconstructed and theoretical sound pressure is derived by Equation (12). The relative amplitude distributions of the reconstructed sound pressure for comparison (200 Hz) are shown in Figure 19. 

In Figure 19, both the PGa-based Patch NAH and CS3C-NAH method can present the spatial *r* and *z* direction positions of the dipole source pairs from the peak and trough of the distributions. However, the relative distributions of sound pressure amplitude reconstructed by the CS3C-NAH method is more accurate than the previous method, and the specific reconstruction errors are shown in Table 5.

**Figure 20:**
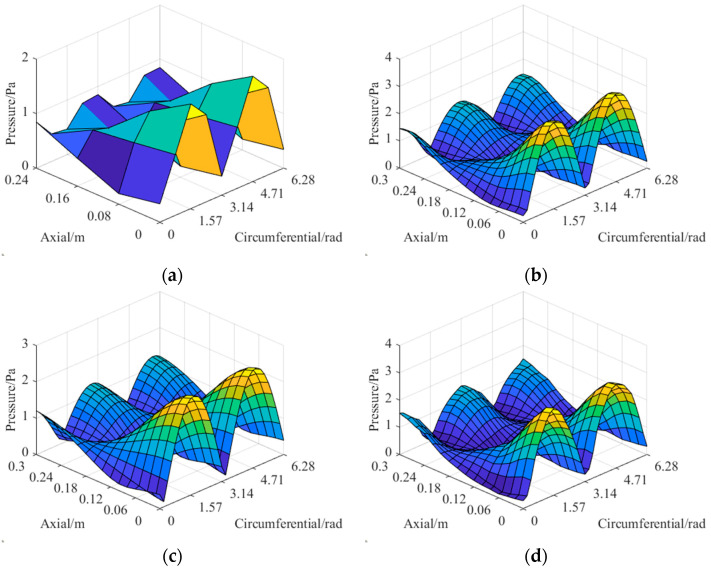
The near-field sound pressure excited by dipole source pair 1: (**a**) represents the sparsely sampled sound pressure on the hologram surface; (**b**) represents the theoretical sound pressure on the reconstructed surface; (**c**) represents the sound pressure reconstructed by the PGa-based Patch NAH method; (**d**) represents the sound pressure reconstructed by the CS3C-NAH method.

**Figure 21:**
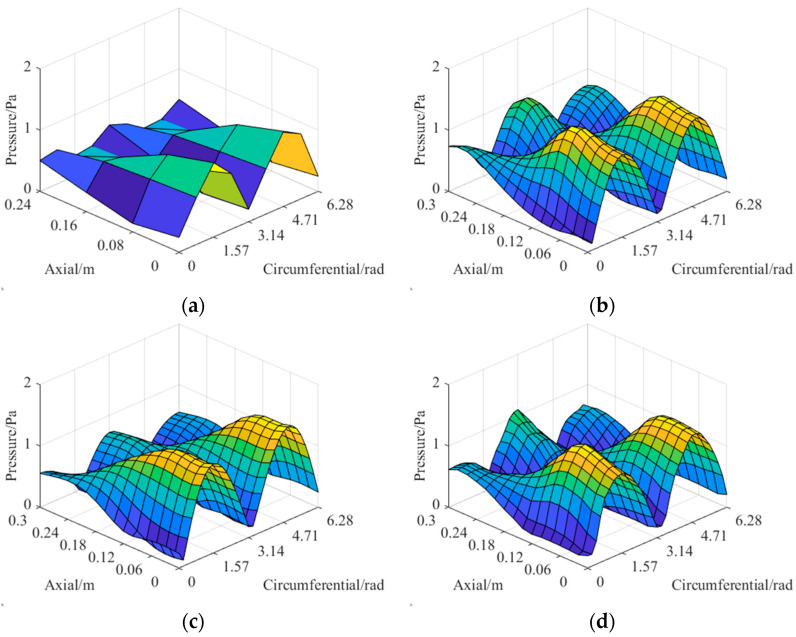
The near-field sound pressure excited by dipole source pair 3: (**a**) represents the sparsely sampled sound pressure on the hologram surface; (**b**) represents the theoretical sound pressure on the reconstructed surface; (**c**) represents the sound pressure reconstructed by the PGa-based Patch NAH method; (**d**) represents the sound pressure reconstructed by the CS3C-NAH method.

**Figure 22:**
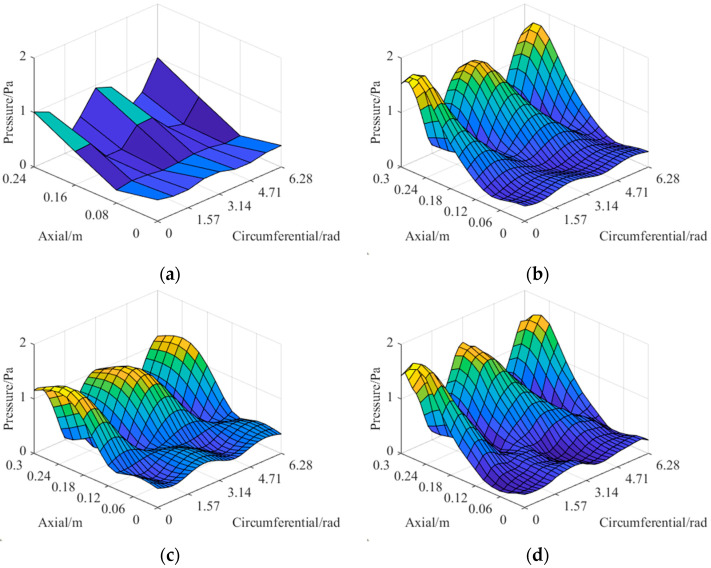
The near-field sound pressure excited by dipole source pair 5: (**a**) represents the sparsely sampled sound pressure on the hologram surface; (**b**) represents the theoretical sound pressure on the reconstructed surface; (**c**) represents the sound pressure reconstructed by the PGa-based Patch NAH method; (**d**) represents the sound pressure reconstructed by the CS3C-NAH method.

It can be seen from Table 5 that the average and maximum of the reconstruction errors of the PGa method are 18.16% and 25.91%, respectively, while those of the CS3C-NAH are 9.52% and 13.47%, which represents a reduction of nearly 50% compared to the PGa method. This may prove that the method is significantly effective. The spatial 3D distributions of the sound pressure data are utilized to illustrate the differences between the PGa-based Patch NAH and the proposed method in detail. These sound distributions are shown in Figure 20, Figure 21 and Figure 22.

It can be seen from Figure 20, Figure 21 and Figure 22 that the PGa-based Patch NAH and the CS3C-NAH method can both accurately locate the sound source position. However, the reconstruction accuracy of CS3C-NAH method is significantly higher, as shown in Figure 19. The quantitative errors for the comparison groups are shown in Table 5. From Figure 20, Figure 21 and Figure 22, the errors of the CS3C-NAH method are 7.11%, 9.75% and 8.71%, while the PGa-based patch NAH has errors of 13.05%, 18.06%, and 20.26%, respectively. At the same time, the CS3C-NAH model can also accurately reconstruct the sound field distributed away from the center of the source. The main reason for which the detail reconstruction accuracy of the PGa-based Patch NAH is inferior to that of the CS3C-NAH is that the spatial Fourier transform and band limit filtering are repeatedly applied to the sound pressure data of the former. Since the sound pressure data on the hologram surface is not a kind of band limit signal in the strict mathematical sense, its spectrum decays by an exponential rule at the bandwidth rather than being cut off directly. Therefore, there will be convergence errors in strict accordance with the processing method for band limit signal. At the same time, due to the influence of noise, the band limit characteristic of the signal is reduced. All of these factors contribute to the errors of the PGa-based Patch NAH algorithm, resulting in the superiority of the proposed CS3C-NAH method.

## 5. Conclusions

From the contradiction between the high resolution of near-field acoustic holography reconstruction and the low sampling rate of measuring, this paper proposed a theoretical method of cylindrical NAH based on deep learning methods to achieve 3D acoustical image super-resolution for sparse holographic sampling. The method is composed of the SAE structure based on 3D-CNN layers and the truncated padding method of CTW, and can use a small quantity of measured data to restore the near-field sound pressure data as accurately as when a high sampling rate is used. It is named CS3C-NAH. The feasibility of the method is verified by numerical example. At the same time, the patch NAH method based on the PGa algorithm in the planar NAH method is introduced into the cylindrical coordinate system and compared with the proposed method. The results show that, for the task of near-field sound pressure reconstruction under the excitation of five random groups of different dipole sound sources pairs, the average and maximum error of the CS3C-NAH method are reduced from 18.16% and 25.91% to 9.52% and 13.47%, respectively, compared with the method based on the PGa theory. This verifies the superiority of the proposed method.

## Figures and Tables

**Table 1:** The position and excitation intensity of the 10 dipole source pairs.

^1^ Position 1 (*r*, *φ*, *z*)	^2^ Intensity 1	^1^ Position 2 (*r*, *φ*, *z*)	^2^ Intensity 2
(50.32, 4.27, 24.69)	3.43	(33.13, 2.59, 197.47)	2.86
(29.18, 3.05, 153.89)	8.95	(4.02, 0.10, 316.61)	9.31
(21.44, 0.22, 140.21)	5.04	(21.33, 4.72, 310.89)	8.00
(50.37, 0.72, 78.23)	5.62	(1.91, 0.39, 316.97)	3.31
(14.01, 0.48, 65.19)	7.89	(23.15, 2.71, 170.81)	7.37
(9.35, 3.82, 20.34)	3.51	(37.50, 3.41, 191.32)	5.67
(37.54, 1.47, 148.08)	2.20	(32.79, 3.83, 227.80)	1.62
(14.57, 0.89, 0.99)	8.23	(55.63, 2.74, 245.98)	4.26
(8.98, 3.33, 29.90)	7.60	(25.17, 0.51, 234.18)	2.94
(15.77, 5.85, 51.92)	8.22	(44.34, 1.78, 260.18)	7.84

Unit of value: ^1^ (10 × 10^−3^ m, rad, 10 × 10^−3^ m), ^2^ ×10^−5^ W.

**Table 2:** The reconstruction errors of the former five source pairs.

Comparison Pairs	(a) with (f)	(b) with (g)	(c) with (h)	(d) with (i)	(e) with (j)
Reconstructed error	11.78%	7.62%	8.06%	11.05%	9.67%

**Table 3:** The reconstruction errors of the latter five source pairs.

Comparison Pairs	(a) with (f)	(b) with (g)	(c) with (h)	(d) with (i)	(e) with (j)
Reconstructed error	7.23%	10.50%	12.28%	9.41%	8.41%

**Table 4:** The position and excitation intensity of the dipole source pairs for comparison.

^1^ Position 1 (*r*, *φ*, *z*)	^2^ Intensity 1	^1^ Position 2 (*r*, *φ*, *z*)	^2^ Intensity 2
(0.34, 4.33, 23.21)	8.55	(1.20, 4.59, 286.71)	6.90
(45.60, 3.34, 10.90)	6.99	(48.45, 3.13, 213.17)	9.27
(35.73, 1.53, 6.77)	7.24	(27.86, 4.32, 232.68)	8.95
(7.98, 0.19, 93.97)	6.84	(11.67, 3.18, 274.21)	2.49
(0.53, 1.06, 150.39)	1.14	(6.15, 0.61, 269.91)	7.88

Unit of value: ^1^ (10 × 10^−3^ m, rad, 10 × 10^−3^ m), ^2^ ×10^−5^ W.

**Table 5:** The comparison of reconstruction errors between different methods.

Dipole Source Pairs	(a)	(b)	(c)	(d)	(e)
PGa error	13.05%	25.91%	18.06%	13.52%	20.26%
CS3C error	7.11%	13.47%	9.75%	8.58%	8.71%

## Data Availability

Data available on request.
